# Tumor reductive therapies and antitumor immunity

**DOI:** 10.18632/oncotarget.18469

**Published:** 2017-06-14

**Authors:** Huiqin Guo, Kangla Tsung

**Affiliations:** ^1^ Department of Thoracic Surgery, Peking Union Medical College Hospital, Beijing, China; ^2^ Department of Surgery, Stanford University School of Medicine, Stanford, California, USA

**Keywords:** cancer reductive therapy, cancer immunotherapy, targeted therapy, tumor antigen, immune checkpoint therapy

## Abstract

Tumor reductive therapy is to reduce tumor burden through direct killing of tumor cells. So far, there is no report on the connection between antitumor immunity and tumor reductive therapies. In the last few years, a new category of cancer treatment, immunotherapy, emerged and they are categorized separately from classic cytotoxic treatments (chemo and radiation therapy). The most prominent examples include cellular therapies (LAK and CAR-T) and immune checkpoint inhibitors (anti-PD-1 and CTLA-4). Recent advances in clinical immunotherapy and our understanding of the mechanism behind them revealed that these therapies have a closer relationship with classic cancer treatments than we thought. In many cases, the effectiveness of classic therapies is heavily influenced by the status of the underlying antitumor-immunity. On the other hand, immunotherapies have shown better outcome when combined with tumor reductive therapies, not only due to the combined effects of tumor killing by each therapy but also because of a synergy between the two. Many clinical observations can be explained once we start to look at these classic therapies from an immunity standpoint. We have seen their direct effect on tumor antigen *in vivo* that they impact antitumor immunity more than we have realized. In turn, antitumor immunity contributes to tumor control and destruction as well. This review will take the immunological view of the classic therapies and summarize historical as well as recent findings in animal and clinical studies to make the argument that most of the cancer treatments exert their ultimate efficacy through antitumor immunity.

## INTRODUCTION

The evolution of cancer therapy is diverse, and continues to be expanded. Tumor reductive therapies include classic therapies (e.g. surgery, chemotherapy, and radiation therapy), modern local and systemic treatment modalities, (e.g. radiation frequency ablation (RFA), local transarterial chemoembolization (TACE), high intensity focused ultrasound (HIFU)), and drugs that target specific molecules in the cancer cells). All cancer reductive therapies have only one objective: to reduce tumor burden through direct killing of tumor cells. A new category of cancer treatment, immunotherapy, emerged in the last few years and they are categorized separately from cytotoxic treatments (chemo and radiation therapy). The most prominent examples include cellular therapies, such as lymphokine-activated killer (LAK) and chimeric antigen receptor T cells CAR-T) and immune checkpoint inhibitors, such as anti-program cell death (PD)-1 and cytotoxic T lymphocyte antigen (CTLA-4). Other modes of tumor destruction have come into clinical use with time. Examples range from older modalities such as radiation frequency ablation (RFA) to novel techniques such as irreversible electroporation (i.e. NanoKnife). With each new piece of oncology knowledge gained, a multitude of questions follow.

## TUMOR REDUCTIVE THERAPY AND ITS CONSEQUENCES

### The role of surgery in tumor reductive therapies

Our initial hypotheses of how tumor reductive therapies work were based solely on their physical or chemical mechanisms; however, with time, these hypotheses became increasingly challenged. For example, surgery is the oldest and most promising mode of tumor reduction that achieves clinical cure in many “early stage” patients. But our recent understanding of tumor mobility confounds this statement. While it was assumed that surgery could eradicate early stage cancer because the primary tumor has not yet metastasized, recent studies with more sensitive analysis show that tumor cell dissemination is a very early event, taking place soon after a tumor becomes vascularized [1, 2]. In most cases, tumors cannot grow over 1-2mm in size without an independent blood supply [3, 4]. By that, one can assume that all primary tumors detectable by imaging and fit for surgery should have already spread to distant sites. This indicates that cancer becomes a systemic disease in a very early stage. How is it that a local therapy such as surgery is able to cure this systemic condition? Indeed, assays have detected metastasized tumor cells in bone marrows of patients with various early stage solid tumors [5–9]. Furthermore, cases of organ transplant recipients, transferred from donors whose solid tumors were cured years before, developing cancer, indicate that tumor bearing is a lifetime event even when patients are clinically “cured” [10–12].

### The relationship between primary tumor and its metastases

Often, tumor metastases do not appear before the removal of the primary tumor. For example, hepatocellular carcinoma rarely presents with extra-hepatic metastases at diagnosis regardless of how large the primary tumor may be (and they are often quite large). Yet extra-hepatic metastases can develop well after removing the primary tumor. This inhibition of metastasis by primary tumor is well known, and the reason might be due to the production of anti-angiogenesis factors such as angiostatin and endostatin, leading to the inhibitory effect of primary tumor on its metastasis [13, 14]. Yet there is no clear evidence for such factors in patients presenting with primary tumors without metastasis in liver cancer [15], and often a negative correlation was seen in other cancers [16]. Furthermore, clinical applications of these protein molecules do not prevent or eliminate metastases [17].

### Cancer chemotherapy and multi-drug resistance (MDR)

While it is understandable that inherent drug resistance is likely correlated with decreased clinical response, the opposite (i.e. sensitivity to chemotherapy drug) is not always true [18]. If chemotherapy is purely direct toxicity on tumor cells, then one would expect that the higher the drug dose, the better response (regardless of patient status); this is simply not always the case in clinical observations and individual patient response to chemotherapy is often unpredictable [19, 20]. A given drug's clinical efficacy varies greatly among patients bearing similar tumors (e.g. adenocarcinoma of the lung). A patient who fails responding to one drug may respond to another completely unrelated drug. But once the tumor acquires drug resistance, its response to all other drugs decrease significantly. Development of drug resistance has been explained by molecular mechanisms such as proteins of the multiple drug resistance (MDR) gene family, yet there is no clear evidence from clinical samples to verify the overwhelming population of chemotherapy -resistant tumor cells. In fact, studies comparing tumor samples from pre- and post-development of so-called chemotherapy resistance consistently find little change of cellular sensitivity to *in vitro* drug testing [21–24]. In addition, means of reversing multiple drug resistance have been developing for years but have not made any significant clinical progress [25, 26], challenging whether this explanation is the true mechanism of acquired chemotherapy resistance.

On the other hand, some of the local treatment modalities seem to have systemic effects as well. Two such examples are the abscopal effect of radiation therapy and the RFA down-staging strategy for the treatment of liver cancers before liver transplant. In the first example, radiation treatment of tumor in one location could cause regression of another distant tumor [27–31]. In the second example, treatment of tumor nodules in a diseased liver by RFA followed by removal and replacement with a non-diseased liver prevented tumor recurrence post-transplant [32]. This practice principle cannot be explained solely by tumor burden reduction to the “allowable” tumor size by pre-transplant criteria – regardless of size, the entire tumor in the diseased liver is removed completely during the liver transplant. In fact, research models indicate that RFA may actually promote residual and distant tumor progression due to generation of local wound-healing factors [33]. The contrasting findings indicate that local therapies may not be as local as initially assumed; rather their mechanisms must be further elucidated to optimize treatment.

Altogether, the above phenomena increasingly suggest that the classic tumor reductive therapies (i.e., by reducing tumor burden) may not work as we previously thought. There are some other factors at play that we do not see. At least one of them is antitumor immunity.

## THE OLD AND NEW CANCER IMMUNOTHERAPIES

The history of tumor immunotherapy extends beyond all other classic tumor reductive therapies except for surgery. The dream of treating cancer by activating one's own immune system has continued to linger, but it was not accepted into standard cancer care until most recently. Immunotherapy, as it is called in modern term, was always relevant to never-ending reports of spontaneous tumor regression, albeit rare [35, 36]. It is these observations that encourage the curious minds to try to duplicate the miracles [37].

### Immunotherapy using viruses and bacteria

Many biological substances ranging from infectious viruses and bacteria to their cellular components have been tested in cancer patients [38–40], some with striking results. The best known (but not necessarily the earliest) example of immunotherapy is that of Coley's Toxin in the late 1890's [34]. Since the identification of lipopolysaccharides (LPS or endotoxin) as the true active ingredient of Coley's toxin in the 1940’s, scientists have tried to pinpoint its mechanism. The subsequent description of a LPS-induced blood factor that can cause tumor necrosis [41, 42] fanned great enthusiasm in clinical application. It drove immunology into its modern age via the molecular cloning technique initially intended to produce tumor necrosis factor (TNF), interferon-gamma, and IL-2. Cytokines were discovered and created in mass quantity and tested in clinical trials against cancer, hoping to duplicate the miracles of Coley's Toxin. But when pure TNF was made available for clinical use, we did not have a wonder drug; instead, more mysteries ensued [43]. For example, cachectin, a well-known factor that was associated with cachexia, was found to be the same molecule as TNF [44] as confirmed by clinical experiment [45]. How could a cytokine that is highly toxic and associated with the most deleterious effect of late stage cancer death be the factor that accounts for Coley's Toxin effect? If not, what are the alternative explanations of the antitumor activity of endotoxins? Furthermore, effects of most of the so-called Biological Response Modifiers (substances that activate host antitumor immunity) have been observed in patients, but vary greatly [46]. This variation was observed in those early Coley's trials using his toxin [40]. This elicits further questions as to what factor(s) predispose a response to immune stimulation, and whether non-response was due to failed immune activation.

### Immunotherapy using vaccines and T cells

Alternative perspectives of immunotherapy arise from other therapeutic strategies, such as tumor vaccines and T cell modulation. With sensitive assays and the precise knowledge of antigens, we saw that modern tumor vaccines did activate specific immune responses in patients [47, 48]. However, the lack of significant overall clinical efficacy triggered tumor immunologists to question this approach [49, 50]. Yet, the belief that the immune system has the power to eradicate cancer was sustained due to occasional outliers of extreme efficacy. For example, though the number of patients was low, recombinant human IL-2 was able to produce dramatic antitumor response in a few patients [51]. The *in vitro* expansion of tumor-infiltrating T cells from cancer patients followed by re-infusion has resulted in clear clinical responses in some patients [52–55]. But the manipulation of an individual patient's immune system does not yield the same results as that of another. This has been the enigma all throughout the history of tumor immunology. Today, no one argues against the potential of antitumor immunity, but the reliability of it. The various efforts to activate one's own immune system to fight cancer, ranging from the amateur approach of Coley's toxin to highly sophisticated cancer vaccine and tumor-specific T cells, have not yet granted us the key to perfect immunotherapy.

### Immunotherapy using immune checkpoints

The recent fanfare for the immune checkpoint therapy, signified by anti-PD-1/PD-L1 antibodies, represents another wave of enthusiasm we have repeatedly seen for cancer immunotherapy. We have added another ammunition in our fight against cancer, one with a novel mechanism that is effective even in patients who have failed all previous therapies. Clinical trials showed that the responses to the new therapies were much broader and lasted longer than past immunotherapies in patients [56–61]. Similar to previous immunotherapy trials, there are several miracles of complete tumor eradication even after the therapy had long stopped [56]. It is this kind of observations that keeps the idea of immunotherapy from dying completely. However, the same question is posed on the variable range of patient responses. The indubitable activation of antitumor immunity in this way (without assistance from any other tumor reductive therapies), tumor regression during the clinical response, followed by gradual loss of efficacy and subsequent tumor relapse in many patients are all events that continue to be observed as a natural course of disease treatment despite persistent therapy [61]. How does the tumor overcome the drug suppression of tumor immunity and halt regression? Furthermore, while it is simply known that the therapy works by PD-L1 inhibition, there are still mysteries shrouding the complete picture of this mechanism. This class of drugs works by removing the blockade of antitumor immunity through PD-L1 expression, similar to removing the brakes on a downward moving truck. That means the truck was already gaining momentum on the slope before it was stopped; antitumor immunity was coexistent with the tumor in order for immune checkpoint therapy to work. If so, why didn't we see it before? How did this antitumor immunity emerge initially and what possible nurturing effect could it have had on the tumor? While the current state of cancer immunotherapy is no panacea, answers to these questions may help us get slightly closer to just that.

## CONCOMITANT ANTITUMOR IMMUNITY: AN INVISIBLE ASPECT OF CANCER MANAGEMENT

### Existence of concomitant antitumor immunity

Unless the antigen is known, specific antitumor immunity is not directly measurable, but that does not disprove its existence. If there was no pre-existing antitumor immunity, how could removing its inhibition have worked as a successful therapy? In this case, the presence of a concomitant antitumor immunity is inferred rather than directly detected. Animal tumor models have long demonstrated this in as early as the 1950s. Many experiments have established that the presence of one tumor, when inoculated into a distant physiological site, prevented the growth of the tumor in the remote site [62–66]. Since this phenomenon was found to depend on host T cells and was tumor-specific [67], it was deduced that the induction of host antitumor immunity prevented the second tumor from grafting. Indeed, subsequent studies have shown that immune cells from tumor-bearing mice could be transferred into another naive mouse, and the recipient was able to reject grafting of the same tumor. By carrying out this transfer at different time points during tumor progression, the initiation and decline of antitumor immunity in the tumor-bearing host was reported [68]. The antitumor immunity subsides in the tumor-bearing host, never fully eliminated. It becomes activated when certain treatments are performed on the tumor-bearing mice. For example, antitumor immunity is “restored” when the tumor is removed by surgery; in several studies, the tumor-excised mice resisted when re-challenged with the same tumor [69–71]. Otherwise, antitumor immunity often stays dormant, co-existing with the progressing tumor.

Other studies have also demonstrated that this concomitant antitumor immunity needs to be in place for certain therapies to work. For example, after the identification of TNF, scientists thought that this factor was the explanation of Coley's toxin because it was the single component that was responsible for the antitumor activity in animal models [72, 73]. However, TNF alone could not duplicate the antitumor activity of LPS despite the fact that it is able to produce tumor necrosis. In order for the tumor to regress completely after administering LPS, the tumor-bearing mice needed to have concomitant antitumor immunity [74]. The same was true when these mice were cured by a combination of chemotherapy and LPS [75].

### The facts about concomitant antitumor immunity

Despite these findings from animal studies, concomitant antitumor immunity has not been accepted into considerations for designing clinical treatments in cancer patients. There are a few reasons for this. First, there is a general belief that human cancers are not immunogenic, thus do not carry concomitant antitumor immunity. This concept was derived from an animal study in which “spontaneous” tumors (i.e., tumors arising naturally rather than induced by chemical carcinogens) tend to be less immunogenic or immuno-stimulatory [76]. Since all human tumors arise spontaneously, it was argued that human cancers are not immunogenic. As such, animal models in which antitumor immunity is a critical component are often considered unrealistic or not closely related to human cancers [77].

The second reason may be that this concomitant antitumor immunity in patients is immeasurable by current assay standards. In animal models, simply removing T cells in the host may reveal the effects of antitumor immunity. The tumor tends to progress more rapidly than the controls [78]. It may also be measured by taking T cells from the host to test their reactivity *in vitro* such as by direct tumor cell killing using cytotoxic T lymphocyte (CTL) assay or measuring cytokine release [79]. Finally, concomitant antitumor immunity is also induced by challenging a tumor-bearing host with the same tumor, or transferring the tumor-bearing host spleen cells to a naive host, followed by tumor challenge and protection assays [80]. But these assays are impractical in human cases. *In vitro* reactivity of tumor-infiltrating T cells (TIL) has been demonstrated [79], supporting the presence of concomitant antitumor immunity in human patients. But these assays are technologically challenging and cannot be performed on every patient.

**Figure 1:**
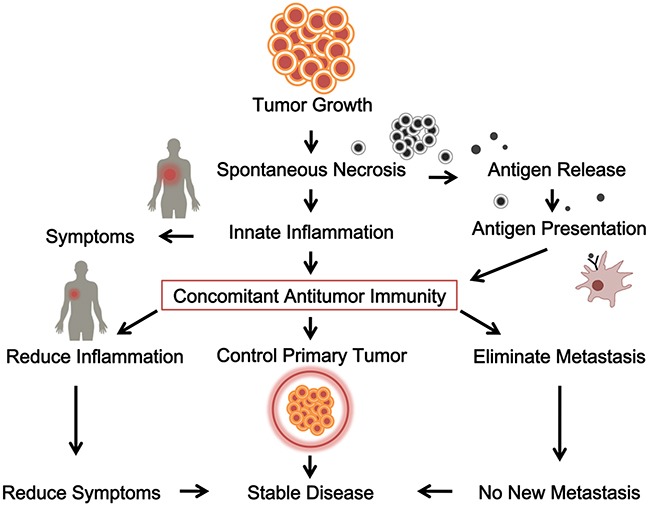
The initiation, establishment and function of a concomitant antitumor immunity in cancer patients Tumor growth releases antigen and induces innate inflammation, stimulates concomitant antitumor immunity, which contributes to control the primary tumor and eliminates metastasis.

Another way that supports concomitant antitumor immunity is the existence of T cell infiltration in tumors from surgery. It has been repeatedly reported that significant T cell presence in solid tumors from surgery correlates with better post-surgery prognosis [81–90]. What this correlation means is that concomitant antitumor immunity is able to protect against post-surgery cancer metastases. But despite repeated publications of similar results, this conclusion has largely been overlooked by the medical society. One of the reasons is the difficulty or subjectivity of interpreting the findings. T cells are often found in tumor samples, but sometimes are associated with poorer prognosis [91, 92]. In addition, the type of T cells may vary greatly ranging from antitumor T cells [93] to suppressive Treg cells [94–96]. Thus, simply estimating the number of intratumor T cells does not predict post-surgery prognosis in one specific patient; however, the overall trend towards a better prognosis is found for multiple tumors with increased T cell presence [97]. On the other hand, since tumors are where antitumor T cells exert their effects, another way to look for the presence of true antitumor T cells in a given tumor would be to quantify them with the negative correlation between tumor infiltrating T cells and tumor growth in T cell-heavy areas [98]. Despite these findings, no current treatment design is based on this analysis. For example, decision for surgery is made on tumor resectability, not whether the patient will likely have post-surgical recurrence and metastases. Even post-surgery treatments are not based on this hypothesis. Therefore, the situation is that on one hand, we see clear presence of concomitant antitumor immunity in cancer patients; on the other hand we do not know how to use this concomitant antitumor immunity for the benefits of the patients. Figure 1 is a diagram summarizing the initiation, establishment, and function of antitumor immunity in cancer patients.

## IMMUNOLOGICAL VIEWS OF CANCER SURGERY

### Cancer surgery and antitumor immunity

Cancer surgery, once thought to be a simple resection of offending tumors, is intricately related to immunology. The known traditional effects of cancer surgery are as follows: 1) reduction of tumor burden to alleviate symptoms associated with it; 2) prevention of tumor dissemination by removing the source; 3) stimulation of new metastases through wound-healing process. The first two functions are obvious and are the reasons behind many rushed surgeries immediately after diagnosis. The third effect is well-established [99, 100] and is likely the basis for recommendations against surgery on metastatic disease. Early scientists, pressured by the fear of metastasis, assumed that early removal of the primary tumor would prevent cancer cell dissemination. This belief, however, is not supported by facts. Cancer cell dissemination is an early and continuous process that takes place soon after a tumor forms independent blood supply through angiogenesis (2). This complex process involves multiple enzymes and growth factors to facilitate individual cancer cells to move out of the circulation and settle down in remote tissues. This is only half of the metastatic process established; the other half requires the cells to produce sufficient factors to attract blood supply through angiogenesis (3). As such, it is a highly variable process among different tumors, even among disseminated tumor cells from the same primary tumor.

However, the tumors found at the point of clinical diagnosis, especially those that already induce symptoms, have most likely spread into circulation (blood and lymphatics) and established dormant or active micro-metastases at distant organs. Several sensitive assays have found circulating tumor cells in almost all patients at diagnosis of solid tumors [6]. This is also consistent with the observation of tumor-containing organs from clinically cured cancer patients who donated their organs many years after cancer surgery [11]. These findings shape the argument that a clinical cure by surgery is not only due to the tumor being contained in one area, nor is it due to the tumor's lack of ability to metastasize. How does surgery, a local therapy, cure cancer, which is systemic by nature? The clue comes from the potential role of concomitant antitumor immunity. Animal studies have shown that the presence of antitumor immunity is able to prevent establishment of tumor metastasis [78]. The positive correlation between T cell infiltration and post-surgical disease-free survival suggests that this also takes place in human cancer. Perhaps cancer survivors lived without recurrence not due to the complete surgical resection of the tumor, but rather due to a residual tumor preventing further cancer metastasis with its corresponding antitumor immunity.

### Cancer surgery and immunological memory

Each individual patient's immunity has evolved alongside its target antigens. While the immunity is enhanced with greater antigen levels, it also contracts according to the reduction of its target antigen [101]. The critical aspect is what happens when the antigen is cleared. The formation of immune memory requires the antigen clearance [102]. During a course of infection, successful clearance of the antigen leads to the establishment of immunological memory for that specific antigen. This is the basis for immunization with vaccines. Low-level antigen persistence prevents memory formation and promotes immune exhaustion or tolerance [103, 104]. When these rules are applied to immunity to tumor antigen, we can explain why complete removal of all visible tumor burden (excluding dormant tumor deposits) is critical [66, 105]. Surgery, compared to other forms of tumor reduction therapy, is more suitable for achieving this goal in cases where no established metastases are present. Incomplete tumor resection would create a situation of antigen reduction but not clearance, thus inducing the antitumor immunity to shrink without being able to form a memory mechanism. As a result, the antitumor immunity wanes and becomes ineffective in preventing future metastases [64, 105]. This explains why incomplete cancer surgery is often deleterious than beneficial and underlines the need for complete tumor resection as indicated by cancer surgery guidelines [106]. On the other hand, surgery as a means to reduce tumor burden benefits a patient's antitumor immunity under certain conditions. For example, under the balance of small antitumor immunity against a large tumor burden, the immunity will likely become exhausted simply because of the overwhelming antigen load [107]. In such situations, surgery can help to significantly tilt the balance toward better disease control due to the pre-surgery concomitant antitumor immunity that needs to be activated [108–110]; otherwise, the antitumor immunity will likely contract with antigen reduction and the patient will lose protection against recurrence and metastases [64]. This explains some of the cases where known incomplete surgery still resulted in disease-free survival. In such situations, surgeons apply electrocauterization *in situ* that burns and destroys tumor deposits, similar to a microwave or radiofrequency-induced ablation causing in situ release of tumor antigen, coupled with inflammation to present the tumor antigens to further activate antitumor immunity. It is through these immunological impacts that make surgery a means to cure a systemic disease.

**Figure 2:**
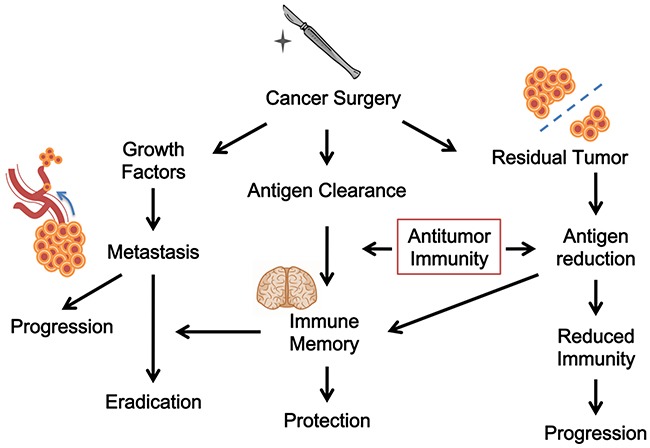
The immunological views of cancer surgery Surgery may cause antigen clearance and antitumor immunity. It could also be tumor stimulatory. Surgery may have different outcomes because of the interaction of various effects.

As such, the most critical prerequisite for surgery to be effective is the presence of concomitant antitumor immunity. Without it, surgery would result only in local but not systemic tumor control. In fact, without the help of concomitant antitumor immunity, surgery alone may be tumor stimulatory as the research models demonstrate [64, 100], explaining many immediate post-surgical appearances of metastases that were not seen before surgery. In this regard, since tumor resection impacts antitumor immunity by reducing or clearing antigens and achieves curative efficacy through it, we can view classic cancer surgery as a form of immunotherapy. Figure 2 depicts the interaction of various effects of cancer surgery and the possible outcome.

## IMMUNOLOGICAL VIEWS OF OTHER TUMOR REDUCTIVE THERAPY

### Chemotherapy and antitumor immunity

Chemotherapy is another major tumor reductive therapy. Its mechanism is thought to be through cellular toxicity. While it is true that chemotherapy drugs are cytotoxic to cancer cells, the antitumor efficacy, however, may not be attributed entirely to this direct cytotoxicity. In animal models where the difference of efficacy in the presence and absence of host antitumor immunity were compared, chemotherapy was found to be more effective in the presence of immunity [111–113]. More recent studies have confirmed these early findings [114–116] while also detailing the molecular parts of chemotherapy-induced immune activation [117]. When chemotherapy drugs eradicate tumor cells, the cells release antigens via specific tumor death (necrosis or apoptosis) that are detected by antitumor immunity. Because every tumor and host HLA combination is unique, not every tumor death by a given drug will lead to the same antigen release, even with the same drug, same tumor type, and same tumor death mechanism. This variation has already been reported in different animal models arguing whether it is better to induce a necrotic cell death or apoptosis [118, 119]. In most of these studies, the requirement of a pre-existing antitumor immunity (concomitant immunity) has been ignored, presuming that as long as tumor cells die an “immunogenic death”, antitumor immunity will be activated. But this is unlikely true in that *de novo* activation of antitumor immunity may not be possible and a pre-existing antitumor immunity is necessary [115].

Since not every patient possesses the same conco-mitant antitumor immunity, there is an unpredictability of activation of antitumor immunity. In patients who do not carry concomitant antitumor immunity regardless of the way in which the tumor cells die and release antigens, there would be no activation of immunity due to the lack of responders. Since antitumor immunity may contribute significantly to the overall chemotherapeutic efficacy [113, 115], its presence or absence and the diverse ways of antigen release will likely cause significant variation among patients with the same tumor treated by the same drugs. This in turn may explain the observed variability and unpredictability during cancer chemotherapy. By the same principle, this explanation also postulates that there would not be a single drug that will give consistent responses as long as participation of antitumor immunity is involved. This hypothesis is supported by the observations made with classic chemotherapeutic drugs thus far.

### Targeted therapy and antitumor immunity

**Figure 3:**
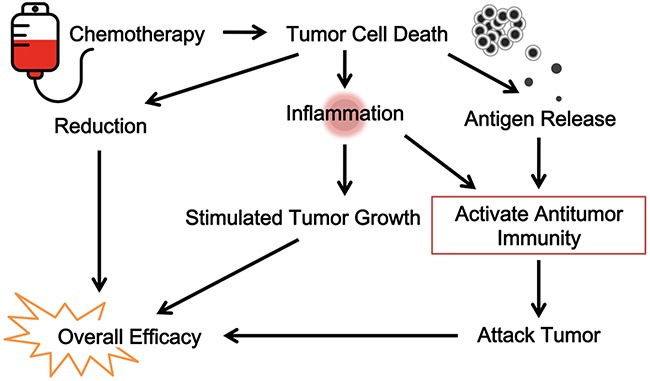
The immunological view of chemotherapy Classic cytotoxic drugs and modern targeted therapy contributes to antitumor immunity. Chemotherapy is, in essence, immunotherapy.

However, recent drugs of targeted therapy, such as small molecular drugs inhibiting certain tumor proliferation-associated receptor kinases (e.g., EGFR) are the exceptions to this rule. Patients who have certain EGFR mutations respond well to targeted inhibitor drugs [120]. This would argue that such a drug-induced response is not dependent on antitumor immunity. Indeed, in most cases, there are no obvious signs of contribution by antitumor immunity. One pattern of response by this immunity is durability, a continued response long after therapy cessation [121]; this is not the case in most patients taking EGFR inhibitors. Symptomatic response and tumor relapse are often immediate upon initiating and terminating these drugs, respectively. The quick response to targeted therapy is not tumor-burden size dependent, but is likely a result of immediate suppression of tumor-induced local inflammation. This is consistent with the known mechanism of apoptosis-induced killing of tumor cells by these drugs [122, 123]. It is likely that continued tumor apoptosis suppresses rather than stimulates adaptive immunity [124]. But this is not absolute truth; in rare cases with large tumor burdens, even these targeted drugs may induce antitumor immunity (our own observation). The differences in response patterns between classic cytotoxic drugs and modern targeted therapy seem to support the participation of antitumor immunity as a major factor behind chemotherapy, thus arguing that this therapy is, in essence, immunotherapy. Figure 3 is a diagram of this view.

### Radiation therapy and antitumor immunity

The same may be true for classic radiation therapy. The abscopal effect of radiation is a historically consistent clinical observation where treatment of one tumor site induces responses in other distant sites [28, 30, 31, 125]. This effect is shown to be the result of activation of antitumor immunity in animal model and in clinical trials [126, 127]. Since killing tumor cells may result in local inflammation and antigen presentation, it is expected that other local treatments may also activate antitumor immunity. One such example is radiofrequency ablation (RFA). In animal models, RFA has been shown to activate antitumor immunity [128–130]. This provides a good explanation for its application in liver cancer treatment via transplant [131]. Guidelines for liver transplant require that the primary tumor be limited in size because larger tumor nodules correlate with high cancer recurrence following liver transplant. In cases where stable reduction of primary tumor size using RFA could be achieved, subsequent liver transplants had significantly reduced recurrence rate [132]. This long-term post-transplant effect cannot be explained solely by tumor nodule reduction perse, since the diseased liver is removed entirely during transplant. Disease relapse is due to re-establishment of tumor nodules by previously disseminated tumor cells outside the diseased liver; thus prevention or elimination of these newly established tumor metastases is the mechanism of disease control. From an immunotherapy point of view, RFA treatment may activate pre-existing antitumor immunity through inflammation and antigen release. This is the factor behind the control of metastases after a cancerous liver is severed. Following the RFA, there is a stable reduction of tumor burden [133].

Animal experiments show that RFA may also actually stimulate intra-hepatic metastases [33], yet this is not always observed in clinical practice. There is a bigger factor at play in the human physiology. The stable reduction of tumor burden by RFA suggests that antitumor immunity is activated, controlling tumor progression. Indeed, only those patients with stable tumor reduction achieved satisfactory prognosis following liver transplant [134]. RFA is therefore also a test to detect the success of immunity activation. It acts as a form of tumor immunotherapy by concurrently enhancing patient's own immunity and subsequently controlling tumor metastases post-transplant.

## CONCLUSIONS

Classic cancer therapies have been manipulated into various regimens to achieve superior efficacy over nearly a century; but the quiet, incremental breakthroughs in chemotherapy do not result in a glorious outcry by the media every time, or by the patients who benefit from them. We have come to expect instant gratification and consistent remedy from our modern medicine. Yet these therapies cure as many times as they fail. This is the major reason that they are much less appreciated than some of the newer developments (immune checkpoint therapy, for example) although it is highly debatable whether the novel treatments can entirely replace the older ones. These classic therapies all work through activation or preservation of antitumor immunity. The variation seen in patient responses are often due to each patient's underlying immune status rather than the direct impact of the therapies themselves. In essence, these are immunotherapies.

The status of antitumor immunity in a given patient is determined by many factors and is likely to be unique to each patient. This predisposes each cancer patient to a unique pattern of responses to a commonly applied therapy. For example, a patient with decent concomitant antitumor immunity before surgery should be able to achieve good prognosis with post-surgical, immunity-mediated protection against future metastasis. With tumors such as breast cancer, a total mastectomy may not require post-surgical chemotherapy. On the other hand, patients without sufficient antitumor immunity before surgery should receive chemotherapy to prevent surgery-induced metastases. In these cases, it is vital to determine the status of host antitumor immunity. Current state of clinical testing does not allow measurement of specific antitumor immunity. Therefore, future developments are needed to derive biomarkers that accurately determine a patient's antitumor immunity status.

Presently, physicians could rely on specific clinical clues to make judgments. For example, a patient with inflammation-induced symptoms that subside naturally under general care is an indication of innate immunity activation, with possible establishment of adaptive antitumor immunity. This could be supported by tests that range from the changes of tumor markers to tumor site metabolism imaging with PET-CT. The appearance of enlarged lymph nodes without metabolic activity under PET-CT may indicate a history of metastases and subsequent control (eradication or suppression) by concomitant antitumor immunity. Activation of this immunity by chemotherapy or radiation followed by surgery may provide a clinical cure. Conversely, a patient presenting with a highly active single primary tumor discovered during regular check-up without any sign of symptoms (inflammation) has likely not established concomitant antitumor immunity. Although the patient may be a good candidate for surgery, the lack of antitumor immunity will not be able to provide post-surgery protection against future metastases. Therefore, when such metastases later arise, the patient will have a bleak outlook for all other therapies.

The abundant animal studies and cases discussed in this review are not only theoretical examples; the clinical evidence is presented to us everyday. Antitumor immunity is intricately woven into every cancer treatment modality in many ways that are still unknown. Future research should focus on solving this puzzle to truly enhance patient's immunity for cancer cure.
